# Multicenter Analytical Comparison of Automated Quantitative Assays Versus Line Immunoassay for Anti‐Ro/La Antibody Detection in Primary Sjögren’s Syndrome

**DOI:** 10.1155/jimr/9771858

**Published:** 2026-06-19

**Authors:** Yu-Lan Chen, Chao-Jun Hu, Lin-Yi Peng, Dong Xu, Wen Zhang, Yan Zhao, Dong-Zhou Liu, Chinese Sjögren’s Syndrome Collaborative Research Group

**Affiliations:** ^1^ Department of Rheumatology and Immunology, Shenzhen People’s Hospital (The First Affiliated Hospital, Southern University of Science and Technology; The Second Clinical Medical College, Jinan University), Shenzhen, 518020, Guangdong, China; ^2^ Department of Rheumatology, Peking Union Medical College Hospital, Key Laboratory of Rheumatology and Clinical Immunology, Ministry of Education, Peking Union Medical College and Chinese Academy of Medical Sciences, Beijing, 100730, China, cams.ac.cn; ^3^ National Clinical Research Center for Dermatologic and Immunologic Diseases (NCRC-DID), Beijing, 100730, China

**Keywords:** chemiluminescence assay, diagnostic performance, line immunoassay, multiplexed bead immunoassay, Sjögren’s syndrome

## Abstract

**Objective:**

This multicenter study aimed to analytically compare the performance of automated quantitative assays—chemiluminescence immunoassay (CLIA) and multiplexed bead immunoassay (MBI)—for detecting anti‐Ro/La antibodies in Chinese patients with primary Sjögren’s syndrome (pSS), compared to line immunoassay (LIA) that is most commonly used in China.

**Methods:**

Based on the Chinese Sjögren’s Syndrome Collaborative Research Group, serum samples from 434 patients with pSS and 100 healthy controls were analyzed using LIA, CLIA, and MBI. Sensitivity, specificity, and qualitative agreement were assessed. Receiver operating characteristic (ROC) analysis was carried out to compare the analytical accuracy among assays in detecting anti‐Ro/La antibodies, and the DeLong test was used to compare areas under curves (AUCs).

**Results:**

High specificity (95% to 100%) was observed in these assays, and LIA demonstrated the highest sensitivity for anti‐Ro60 (92.6%) and anti‐Ro52 (89.2%). CLIA and MBI exhibited comparable sensitivity to LIA for anti‐La (50.9% vs. 50.2% vs. 48.2%). Qualitative agreement among assays was good for anti‐Ro60 (*κ*: 0.84–0.93) and moderate for anti‐La (*κ*: 0.74–0.86). MBI achieved the highest AUC values for anti‐Ro60 (0.980), anti‐Ro52 (0.970), and anti‐La (0.935), outperforming CLIA and LIA (*p*  < 0.05).

**Conclusion:**

Automated assays (CLIA and MBI) demonstrate high specificity and analytical accuracy for anti‐Ro/La antibody detection in pSS. MBI achieved the highest accuracy, whereas LIA showed superior sensitivity for anti‐Ro60/52 detection. CLIA provided reliable specificity but failed to detect anti‐Ro52 in this study. Collectively, these findings indicate that CLIA and MBI may represent reliable alternatives to LIA, although validation in disease‐controlled cohorts is required before clinical implementation.

## 1. Introduction

Primary Sjögren’s syndrome (pSS) is a systemic autoimmune disease that primarily targets the exocrine glands (thereby causing sicca syndrome) and is associated with possible organ involvement. Antibodies directed against Ro and La autoantigens are the hallmark of pSS and have long been included in the classification criteria for the disease [1–3]. Anti‐Ro antibodies can either be detected alone or concomitantly with anti‐La antibodies; isolated anti‐La positivity is reported in only 2.3% to 7% of patients with Sjögren’s syndrome (SS) [4].

The Ro antigen includes Ro60 and Ro52 proteins, which are associated with small molecules of ribonucleic acid (RNA); they were initially considered to constitute a stable macromolecular ribonucleoprotein complex [5]. However, recent evidence suggests that anti‐Ro60 and anti‐Ro52 antibodies may be independent with distinct clinical implications and that separate detection of the two antibodies is desirable in the clinical diagnostic setting [6, 7].

Anti‐Ro and anti‐La antibody detection constitutes a mandatory criterion for classification of pSS, especially in patients without positive salivary gland biopsy results [1–3]. Autoantibodies against Ro and La antigens have been reported to be present in the tear fluid of patients with SS, which is associated with the severity of keratoconjunctivitis sicca [8]. Anti‐Ro60 antibodies are also strongly associated with sensory peripheral neuropathy in pSS [9]. Intriguingly, although anti‐Ro52 antibodies have been considered to lack specificity in connective tissue diseases, including SS, studies on mouse models have shown that Ro52‐induced antibodies are capable of inducing SS‐like disorders [10, 11]. In addition, anti‐Ro52 antibodies have been reported to be associated with a broad spectrum of autoimmune diseases, particularly idiopathic inflammatory myopathy (IIM) [12–14]. In this context, a recent study showed that anti‐La antibody‐positivity in pSS is significantly associated with hypergammaglobulinaemia, rheumatoid factor positivity, and lymphoma [15]. Therefore, defining the anti‐Ro and anti‐La profile has diagnostic and prognostic implications in pSS.

Various methods are used to detect anti‐Ro and anti‐La antibodies, such as double‐immunodiffusion, counterimmunoelectrophoresis, immunoblotting, line immunoassay (LIA), enzyme‐linked immunosorbent assay (ELISA). LIA is the most common method used in China, while ELISA is the most frequently used in Western countries [16]. Owing to advancements in immunological detection technology, conventional diagnostic assays are being replaced by automated quantitative and multiplex assays. Chemiluminescence assay (CLIA) and multiplexed bead immunoassay (MBI) have gradually emerged as alternatives for routine autoantibody testing. However, no assay has been recommended as the gold standard for the detection of anti‐Ro and anti‐La antibodies. In addition, the performance of the new assays has not been well evaluated in Chinese patients with pSS. The present multicenter study, therefore, aimed to analytically compare the performance characteristics of two automated quantitative immunoassays, namely, CLIA and MBI, in patients with pSS, based on the Chinese Sjögren’s Syndrome Collaborative Research Group; it also aimed to compare their performance with that of LIA in the detection of anti‐Ro and anti‐La antibodies.

## 2. Methods

### 2.1. Study Patients

A total of 434 patients from 21 tertiary university teaching hospitals, who were diagnosed with pSS between June 2019 and October 2019, were recruited in this study. All patients fulfilled the 2016 American College of Rheumatology/European League against Rheumatism classification criteria, the 2012 Sjögren’s International Collaborative Clinical Alliance preliminary criteria, or the 2002 American–European Consensus Group criteria for SS [1–3]. Patients demonstrating overlap with other systemic autoimmune diseases (such as systemic lupus erythematosus [SLE], rheumatoid arthritis, dermatomyositis, or scleroderma); having human immunodeficiency virus, treponema pallidum, or other serious infections; and having malignancies were excluded from this study. Pregnant or lactating women were also excluded. A total of 100 healthy controls were included concurrently. Participants gave their written informed consent, and this study was approved by the ethics committees of the leading centers (Shenzhen People’s Hospital, identifier: YKLS2019‐15‐01; Peking Union Medical College Hospital, identifier: JS‐2038) and complied with the Helsinki Declaration.

### 2.2. Autoantibody Detection

Serum samples from individuals with pSS and healthy controls were obtained and stored at −80°C until analysis. Three different methods were applied to detect anti‐Ro and anti‐La antibodies; these included LIA, CLIA, and MBI. All serological testing was performed centrally at the Key Laboratory of Rheumatology and Clinical Immunology of the Chinese Academy of Medical Sciences. The characteristics of the different assays are shown in Table 1.

**Table 1:** Characteristics of the assays in detecting antibodies.

Assay	Antigen	Antigen resource	Supplier	Qualitative/quantitative	Cut‐off value	Upper limit value
LIA	Ro60	Native	Euroimmun	Qualitative	Positive: OD ≥ 16	+∞
	Ro52	Recombinant	Euroimmun	Qualitative	Positive: OD ≥ 16	+∞
	La	Recombinant	Euroimmun	Qualitative	Positive: OD ≥ 16	+∞
CLIA	Ro60	Recombinant	Shenzhen Yahuilong Biotechnology	Quantitative	Positive: ≥20 AU/mL	200 AU/mL
	La	Recombinant	Shenzhen Yahuilong Biotechnology	Quantitative	Positive: ≥20 AU/mL	400 AU/mL
MBI	Ro60	Recombinant	Shanghai Toujing Life Technology	Quantitative	Positive: ≥20 AU	+∞
	Ro52	Recombinant	Shanghai Toujing Life Technology	Quantitative	Positive: ≥20 AU	+∞
	La	Recombinant	Shanghai Toujing Life Technology	Quantitative	Positive: ≥20 AU	+∞

Abbreviations: CLIA, chemiluminescence assay; LIA, line immunoassay; MBI, multiplexed bead immunoassay; OD, optical density.

#### 2.2.1. LIA

LIA was performed with Euroimmun test kits (Euroimmun AG, Lübeck, Germany) and was analyzed using the EUROLineMaster Plus‐A analyzer (Euroimmun AG, Lübeck, Germany). The kit performed simultaneous qualitative assessment of the presence of human IgG autoantibodies against 15 different specific antigens, which were precoated as parallel lines on the membrane strip; these included: nuclear RNP (nRNP), Smith (Sm), Scl‐70, polymyositis‐sclerosis (PM‐Scl), Jo‐1, centromere protein B (CENP‐B), proliferating cell nuclear antigen (PCNA), double‐stranded DNA (dsDNA), nucleosome, histone, ribosomal P protein (P0), Ro60, Ro52, La, and AMA‐M2. The assay was performed in accordance with the manufacturer’s instructions. The cut‐off values are demonstrated in Table 1.

#### 2.2.2. CLIA

CLIA (Shenzhen Yahuilong Biotechnology Co., Ltd.) was performed according to the manufacturer’s instructions using the fully automated iFlash 3000 CLIA analyzer (Shenzhen Yahuilong Biotechnology Co., Ltd.) to detect the specific IgG antibodies separately; the antibodies included anti‐Ro60 and anti‐La, but not anti‐Ro52. The cut‐off value for anti‐Ro60 and anti‐La antibody positivity, as proposed by the manufacturer, was ≥20 AU/mL. The upper limit of the detection range is 200 AU/mL for anti‐Ro60 antibodies and 400 AU/mL for anti‐La antibodies, respectively (Table 1). The kits for CLIA were licensed, commercialized, and available in clinical practice.

#### 2.2.3. MBI

MBI, an automated multiplex immunoassay using flow cytometry, was performed using autoantibody test kits (Shanghai Toujing Life Technology Co., Ltd.) with substrate; goat antihuman IgG specific R‐phycoerythrin‐labeled conjugates were used to detect a panel of IgG antibodies against dsDNA, complement 1q, RNP, Sm, Ro60, Ro52, La, Scl‐70, CENP‐B, PM‐Scl, Jo‐1, PCNA, nucleosome, histone, P0, and AMA‐M2. All operations were performed in accordance with the instrument manual, and the microsphere suspension was analyzed using the fully automated Tellgen Super Multiplex Immunoassay System (TESMI‐F4000). Anti‐Ro60, anti‐Ro52, and anti‐La antibody concentrations of ≥20 Arbitrary Unit (AU) were defined as positive, respectively (Table 1). The kits for MBI were also licensed, commercialized, and available in clinical practice.

### 2.3. Statistical Methods

Data analysis was performed using SPSS 20.0 for Windows (SPSS Inc., Chicago, IL, USA), MedCalc (MedCalc Software, Ostend, Belgium), and online tools (VassarStats: http://vassarstats.net; https://www.graphpad.com/quickcalcs). Quantitative data are expressed as median (Interquartile range, IQR). Categorical variables are expressed as percentages (%). The sensitivity and specificity of the assays were calculated, and results were presented as percentages (%). The McNemar’s chi‐squared test for paired proportions was used to compare sensitivity and specificity between the assays. Pairwise correlations between assay results were assessed using Spearman’s rank correlation, and correlation coefficients (*r*) were reported. Cohen’s *κ* agreement test was used for consistency analysis of different assays; *κ* values of 0.60–0.80, 0.80−0.90, and over 0.90 indicated moderate, substantial, and almost perfect agreement, respectively [17]. Receiver operating characteristic (ROC) analysis was performed to compare test accuracy between different methods, and areas under curves (AUCs) were calculated. The DeLong test was used to compare AUCs [18]; *p*  < 0.05 indicated statistical significance.

## 3. Results

### 3.1. Clinical Characteristics of Patients With pSS

The demographic, clinical, and laboratory characteristics of the patients with pSS are demonstrated in Table S1. Patients were predominantly female (97.4%), with a median age of 49.5 years and disease duration of 24 months. Sicca symptoms were common, while extraglandular involvement was observed in a subset of patients, particularly hematological (35.8%) and interstitial lung disease (ILD, 9.7%). High positivity rates were observed for ANA (93.8%) and anti‐Ro60 antibody (93.6%), and 34.7% of patients had hypergammaglobulinemia; the median EULAR Sjögren’s Syndrome Disease Activity Index (ESSDAI) score was 2.0 (IQR, 0–7.0).

### 3.2. Prevalence of Anti‐Ro and Anti‐La Antibodies in pSS

Three assays, including LIA, CLIA, and MBI, were used to evaluate samples from 434 patients with pSS and 100 healthy controls. The prevalence of anti‐Ro and anti‐La antibodies is shown in Table 2. In addition, the detailed numbers of true‐positive, false‐positive, true‐negative, and false‐negative results for each assay in patients with pSS are presented in Table S2.

**Table 2:** Percentage of positive tests for each assay in patients with pSS and healthy controls ^∗^.

Group	Antibody	LIA	CLIA	MBI
pSS (*n* = 434)	Anti‐Ro60	402 (92.6)	382 (88.0)	367 (85.0)
	Anti‐Ro52	387 (89.2)	—	368 (84.8)
	Anti‐La	209 (48.2)	221 (50.9)	218 (50.2)
Healthy controls (*n* = 100)	Anti‐Ro60	0	0	0
	Anti‐Ro52	5 (5.0)	—	1 (1.0)
	Anti‐La	0	0	1 (1.0)

Abbreviations: CLIA, chemiluminescence assay; LIA, line immunoassay; MBI, multiplexed bead immunoassay; pSS, primary Sjögren’s syndrome.

**^∗^** Data are shown as *n* (%).

In patients with pSS, LIA demonstrated the highest positivity rate for detection of anti‐Ro60 (92.6%) and CLIA showed the highest rate for anti‐La positivity (50.9%). Only 1 (1%) healthy control was found to test positive for anti‐La on MBI. For anti‐Ro52, LIA showed a higher positivity rate (89.2%) in patients with pSS; however, 5% and 1% of healthy controls also tested positive with LIA and MBI, respectively.

### 3.3. Diagnostic Performance Characteristics of Different Assays in pSS

The diagnostic performance characteristics of the three assays are shown in Table 3. For anti‐Ro60 antibodies, LIA, CLIA, and MBI demonstrated specificities of 100% (95% confidence interval [CI]: 95.4%–100%), 100.0% (95% CI: 95.4%−100.0%), 100.0% (95% CI: 95.4%−100.0%), respectively. CLIA and MBI showed significantly lower sensitivity compared with that of LIA (88.0% vs. 92.6% and 85.0% vs. 92.6%, respectively; both *p*  < 0.05). LIA showed the highest negative predictive value (NPV) (75.7%, 95% CI: 67.4%−82.6%) and the lowest negative likelihood ratio (NLR) (0.07, 95% CI: 0.05–0.10).

**Table 3:** Diagnostic performance of the three methods in detecting anti‐Ro and anti‐La antibodies.

Parameter	Anti‐Ro60			Anti‐Ro52			Anti‐La	
	LIA	CLIA	MBI	LIA	MBI	LIA	CLIA	MBI
Sensitivity (%)	92.6^a,b^ (89.6–94.8)	88.0^a^ (84.5–90.8)	85.0^b^ (81.2–88.2)	89.2^b^ (85.8–91.9)	84.8^b^ (81.0–88.0)	48.2^a^ (43.4–53.0)	50.9^a^ (46.1–55.7)	50.2 (45.4–55.2)
Specificity (%)	100 (95.4–100)	100 (95.4–100)	100 (95.4–100)	95.0 (88.2–98.1)	99.0 (93.8–99.9)	100 (95.4–100)	100 (95.4–100)	99.0 (93.8–99.9)
PPV (%)	100 (98.8–100)	100 (98.8–100)	100 (98.7–100)	98.7 (96.9–99.5)	99.7 (98.3–100)	100 (97.8–100)	100 (97.9–100)	99.5 (97.1–100)
NPV (%)	75.7 (67.4–82.6)	65.8 (57.6–73.2)	60.6 (52.7–68.0)	66.9 (58.4–74.4)	60.0 (52.1–67.5)	30.7 (25.9–36.1)	31.9 (26.9–37.5)	31.4 (26.4–36.9)
PLR	+∞	+∞	+∞	17.8 (7.6–41.9)	84.8 (12.1–596.3)	+∞	+∞	50.2 (7.1–353.9)
NLR	0.07 (0.05–0.10)	0.12 (0.09–0.15)	0.15 (0.12–0.19)	0.11 (0.09–0.15)	0.15 (0.12–0.19)	0.52 (0.47–0.57)	0.49 (0.45–0.54)	0.50 (0.46–0.55)

Abbreviations: CLIA, chemiluminescence assay; LIA, line immunoassay; MBI, multiplexed bead immunoassay; NLR, likelihood ratio for a negative result; NPV, negative predictive value; PLR, likelihood ratio for a positive result; PPV, positive predictive value.

^a^Significant difference between LIA and CLIA.

^b^Significant difference between LIA and MBI.

LIA and MBI showed specificities of 95.0% (95% CI: 88.2%−98.1%) and 99.0% (95% CI: 93.8%−99.9%), respectively, for anti‐Ro52 antibody testing. Compared to MBI, LIA demonstrated significantly higher sensitivity (89.2% vs. 84.8%, *p*  < 0.001), a higher NPV (66.9%, 95% CI: 58.4%−74.4%), and a lower NLR (0.11, 95% CI: 0.09–0.15).

LIA, CLIA, and MBI demonstrated sensitivities of 48.2% (95% CI: 43.4%−53.0%), 50.9% (95% CI: 46.1%−55.7%), and 50.2% (95% CI: 45.4%−55.2%), respectively, for anti‐La antibody testing. CLIA showed significantly higher sensitivity than LIA (50.9% vs. 48.2%, *p*  < 0.001); however, no statistically significant differences were observed between LIA and MBI. As summarized in Table 3, no significant differences were observed between LIA, CLIA, and MBI in terms of specificity. The NLRs for LIA, CLIA, and MBI were 0.52, 0.49, and 0.50, respectively.

### 3.4. Qualitative Agreement Among Different Methods

**Figure 1:**
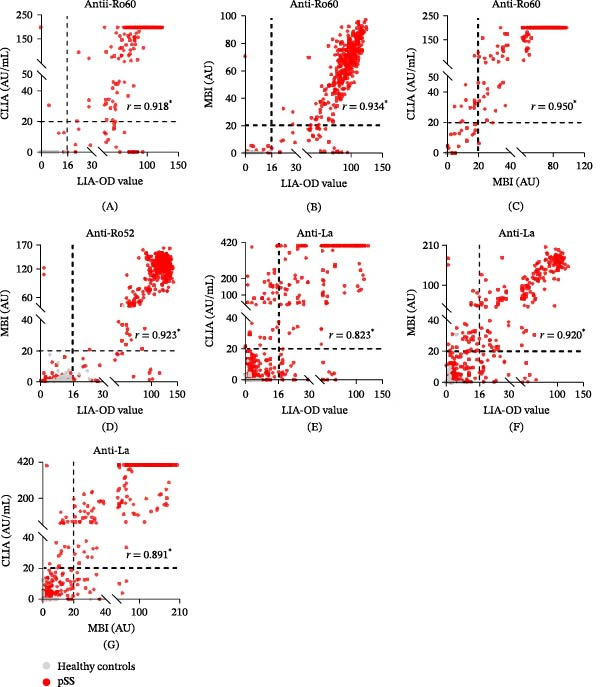
Scatter plot of test results from different assays. Comparison and correlation of anti‐Ro60 (A–C), anti‐Ro52 (D), and anti‐La (E–G) results obtained by LIA, CLIA, and MBI. The analysis included samples from 434 patients with primary Sjögren’s syndrome and 100 healthy controls. Each dot represents an individual sample. Pairwise correlations between assay results were evaluated using Spearman’s rank correlation. Correlation coefficients (*r*) are shown in the corresponding panels. CLIA, chemiluminescence assay; LIA, line immunoassay; MBI, multiplexed bead immunoassay.  ^∗^ *p*  < 0.001 (two‐tailed).

The comparison of test results for anti‐Ro and anti‐La antibodies from different assays is shown in Figure 1. Good agreement was achieved among different assays in detecting anti‐Ro60 (LIA, CLIA, and MBI) and anti‐Ro52 antibodies (LIA and MBI). In contrast, a relatively lower consistency was observed in anti‐La testing between LIA and CLIA or MBI (Figure 1E,F). In addition, pairwise correlations between results obtained by LIA, CLIA, and MBI were assessed. Strong correlations were observed among the assays for the detection of anti‐Ro and anti‐La antibodies (*r* = 0.823–0.950, all *p*  < 0.001).

As demonstrated in Table 4, qualitative agreement between CLIA, MBI, and LIA was calculated in the detection of anti‐Ro and anti‐La antibodies. Good qualitative agreement was observed among the three methods in terms of anti‐Ro60 antibody detection, with substantial to almost perfect concordance across all pairwise comparisons (LIA vs. MBI: *κ* 0.84, 95%CI: 0.79–0.89; LIA vs. CLIA: *κ* 0.89, 95% CI: 0.84–0.93; CLIA vs. MBI: *κ* 0.93, 95% CI: 0.90–0.97); the total percent agreement (TPA) varied between 93.5% (95% CI: 91.0%−95.3%) (LIA vs. MBI) and 97.2% (95% CI: 95.4%−98.3%) (CLIA vs. MBI). Based on the *κ* value, LIA and MBI showed good consistency for anti‐Ro52 detection (*κ* 0.88, 95% CI: 0.83–0.92) with a TPA value of 94.9% (95% CI: 92.7%−96.5%).

**Table 4:** Qualitative agreement between different methods.

Parameter	Anti‐Ro60		Anti‐Ro52		Anti‐La	
	TPA (%)	*κ*	TPA (%)	*κ*	TPA (%)	*κ*
LIA vs. CLIA	95.5 (93.4–97.0)	0.89 (0.84–0.93)	—	—	89.3 (86.4–91.7)	0.74 (0.68–0.79)
CLIA vs. MBI	97.2 (95.4–98.3)	0.93 (0.90–0.97)	—	—	93.3 (90.8–95.1)	0.86 (0.82–0.90)
LIA vs. MBI	93.5 (91.0–95.3)	0.84 (0.79–0.89)	94.9 (92.7–96.5)	0.88 (0.83–0.92)	88.0 (85.0–90.5)	0.75 (0.69–0.81)

Abbreviations: CLIA, chemiluminescence assay; LIA, line immunoassay; MBI, multiplexed bead immunoassay; TPA, total percent agreement.

Good qualitative agreement was also achieved between CLIA and MBI for the detection of anti‐La antibodies (*κ* 0.86, 95% CI: 0.82–0.90); the agreements between the other assays were moderate (LIA vs. CLIA: *κ* 0.74, 95% CI: 0.68–0.79; LIA vs. MBI: *κ* 0.75, 95% CI: 0.69–0.81), with TPA values varying between 88.0% (95% CI: 85.0%−90.5%) and 93.3% (95% CI: 90.8%−95.1%).

### 3.5. Analytical Accuracy of Different Methods

ROC curves and AUC values were determined for each assay to individually evaluate their analytical accuracy. Based on the AUC values, all three assays demonstrated good diagnostic value for pSS. The AUC values for different assays, as derived from ROC analysis, are compared in Table 5.

**Table 5:** Comparison of areas under curves between methods.

Antibody	AUC (95% CI)			DeLong test (*z*‐statistic)/*p*‐value		
	LIA	CLIA	MBI	LIA vs. CLIA	LIA vs. MBI	CLIA vs. MBI
Anti‐Ro60	0.958 (0.938–0.974)	0.956 (0.935–0.972)	0.980 (0.964–0.990)	0.342 *p* = 0.7327	2.768 *p* = 0.0056	4.423 *p* < 0.0001
Anti‐Ro52	0.915 (0.888–0.937)	—	0.970 (0.951–0.983)	—	5.009 *p* < 0.0001	—
Anti‐La	0.859 (0.826–0.887)	0.882 (0.851–0.908)	0.935 (0.910–0.954)	1.412 *p* = 0.1579	4.307 *p* < 0.0001	3.757 *p* = 0.0002

Abbreviations: AUC, areas under curve; CLIA, chemiluminescence assay; LIA, line immunoassay; MBI, multiplexed bead immunoassay.

MBI yielded a significantly higher AUC value (0.980, 95% CI: 0.964–0.990) for anti‐Ro60 testing than CLIA (0.956, 95% CI: 0.935–0.972; z statistic: 4.423, *p*  < 0.0001) and LIA (0.958, 95% CI: 0.938–0.974; z statistic: 2.768, *p* = 0.0056); there were no significant differences in AUC values between LIA and CLIA. MBI (0.970, 95% CI: 0.951–0.983) demonstrated a significantly higher AUC value for anti‐Ro52 detection than LIA (0.915, 95% CI: 0.888–0.937; z statistic: 5.009; *p*  < 0.0001).

For anti‐La testing, MBI achieved the highest AUC value (0.935, 95% CI: 0.910–0.954; *p*  < 0.0001), which was significantly higher than that of LIA (0.859, 95% CI: 0.826–0.887; z statistic: 4.307, *p* < 0.0001) and CLIA (0.882, 95% CI: 0.851–0.908; z statistic: 3.757, *p* = 0.0002). No significant difference was found between AUC values yielded by CLIA and LIA.

## 4. Discussion

This study evaluated the diagnostic performance of automated quantitative assays (CLIA and MBI) in the detection of anti‐Ro and anti‐La antibodies among patients diagnosed with pSS; the study also compared the performance of these assays with that of LIA, which is currently the most commonly used assay in China. Samples were obtained from a multicenter cohort based on the Chinese Sjögren’s Syndrome Collaborative Research Group. The results revealed high specificity (varying from 95% to 100%) in the detection of both anti‐Ro and anti‐La antibodies in patients with pSS. Although the McNemar’s test showed statistically significant difference in terms of sensitivity, moderate to good qualitative agreement was achieved between the assays with *κ* values ranging from 0.74 to 0.93. Notably, good diagnostic values were achieved by all three assays based on the AUC values; MBI yielded the best AUC values for anti‐Ro60 and anti‐La antibody detection in pSS. Therefore, the current study indicates that automated quantitative assays (CLIA and MBI) are viable alternatives to LIA in the detection of anti‐Ro and anti‐La antibodies in patients with pSS.

There is currently no recommended assay for anti‐Ro and anti‐La antibody detection. Different assays have been predominantly adopted to detect anti‐Ro and anti‐La antibodies, including RNA precipitation, counterimmunoelectrophoresis, LIA, ELISA, CLIA, and MBI. The study by Manoussakis et al. [19] has revealed that RNA precipitation assay can be used as the reference method due to the highest sensitivity for anti‐Ro antibody detection. However, the assay is too complex to be applied in clinical setting. Meanwhile, although counterimmunoelectrophoresis appears to be a reliable method due to relatively satisfactory specificity in the detection of anti‐Ro antibodies [19], it can be time‐consuming and high‐cost to detect a vast quantity of samples. Although LIA allows for multiplexed testing with high sensitivity for anti‐Ro antibodies, a relatively low specificity has been reported in previous studies [20]. Another assay, namely, ELISA, was subsequently adopted. However, it may also have low specificity [21, 22]; single reactivity to either Ro60 or Ro52 may therefore be misinterpreted in cases with a mixture of both antigens [6]. LIA is the most commonly used assay in China, while ELISA is the most frequent in Western countries [16]. LIA and ELISA have shown good concordance in the detection of anti‐Ro52 reactivity, with a *κ* value of 0.82 [23]. The landscape of laboratory screening for autoimmune diseases has changed substantially with the development of immunological detection technologies and the introduction of automated quantitative assays, such as CLIA and MBI. A recent meta‐analysis showed that CLIA has better specificity for detection of ANA than indirect immunofluorescence [24]. A good agreement has also been found between CLIA and ELISA in the detection of anti‐Ro (*κ* 0.864) and anti‐La (*κ* 0.735) antibodies [25]. On the other hand, Hanly et al. [26] reported good agreement between MBI and LIA for anti‐Ro detection (*κ* 0.84) and anti‐La (*κ* 0.74). MBI offers definite advantages over conventional techniques in terms of higher throughput, lower test sample volumes, and less dependence on highly skilled operators [27]. In this context, we had recently performed a multicenter study on autoantibody detection in China and found a trend toward the application of automatic and quantitative methods [28]. LIA is a well‐established method in China; however, the performance of CLIA and MBI has not been extensively studied and compared in Chinese patients with pSS. We had therefore performed this multicenter study. To the best of our knowledge, this is the first study to compare the performance of CLIA and MBI with that of LIA based on the Chinese Sjögren’s Syndrome Collaborative Research Group.

This study, which adopted cut‐off values recommended by the manufacturer, revealed high specificity for all three assays, consistent with the similarly high specificity (99%–100%) for anti‐Ro and anti‐La antibody detection reported by Trier et al. [29]. Nevertheless, there were significant differences in sensitivity among the assays in this study. In contrast to the findings of Au et al. [30] in patients with SLE, where MBI outperformed LIA in the detection of both anti‐Ro and anti‐La antibodies, our results indicate a superior or at least comparable performance of LIA relative to MBI for anti‐Ro60 and anti‐Ro52 detection. This discrepancy suggests that assay performance may vary substantially across different disease populations. Similarly, previous studies in patients with pSS reported lower sensitivities of LIA for anti‐Ro60 (69% vs. 92.6%) and anti‐Ro52 antibodies (76% vs. 89.2%) than those observed in our cohort [29], whereas comparable performance between LIA and CLIA for anti‐La detection appears to be consistent across studies (48.2% vs. 50.9%). These partially concordant and partially divergent findings may be attributable to differences in patient characteristics, disease spectrum, and antigen composition. Furthermore, Claessens et al. [31] reported a high sensitivity (91%) of CLIA for ANA screening in pSS, which is not directly comparable to the detection of individual extractable nuclear antigens but nevertheless underscores that assay performance is both antigen‐ and disease‐dependent. Taken together, these observations highlight that the comparative diagnostic performance of assays cannot be generalized across diseases or antibody specificities.

Despite the differences in sensitivity, good qualitative agreement was achieved among assays in the detection of anti‐Ro antibodies; moderate agreement was found for anti‐La antibody detection. In a recent study from Hong Kong, the performance of LIA was compared to that of MBI in the detection of anti‐ENA antibodies among Chinese patients with SLE [30]. The results revealed good agreement between LIA and MBI in the detection of anti‐Ro antibodies (*κ* 0.79) and moderate agreement for anti‐La antibodies (*κ* 0.65), which aligns closely with the agreement profile observed in our cohort. Similarly, Hanly et al. [32] also reported a substantial to almost perfect agreement between LIA and MBI for anti‐Ro and anti‐La testing (*κ* 0.75–0.93) in patients with SLE, further supporting the robustness of interassay concordance for these targets. In addition, Wei et al. [33] reported substantial agreement between LIA and CLIA for ANA testing (anti‐Ro60: 57.14% vs. 61.22% and anti‐La: 11.56% vs. 17.01%, respectively). Our observation that all three platforms exhibit good diagnostic value in pSS is concordant with reports showing high AUC values for MBI in systemic autoimmune rheumatic diseases (AUC 0.814) [34], as well as strong discriminative ability of MBI for individual autoantibodies in pSS, with AUC values of 0.916, 0.909, and 0.856 for anti‐ Ro60, anti‐Ro52, and anti‐La antibodies, respectively [35]. In addition, the robust performance of CLIA observed in our cohort is in line with prior reports showing high overall diagnostic accuracy of CLIA in pSS populations (AUC 0.949) [31]. Collectively, these data suggest that although quantitative sensitivity and AUC values may vary between platforms and study populations, qualitative agreement and overall diagnostic utility remain largely consistent across assays, reinforcing the concept that comparative assay performance is influenced by both disease context and antigen specificity.

Most CLIA test kits currently used in China are designed solely for the detection of anti‐Ro60 antibodies and do not include anti‐Ro52 antibody testing. CLIA kits used in this study, therefore, focused on Ro60 reactivity and did not screen specifically for Ro52. Actually, anti‐Ro52 antibodies are generally considered to have limited disease specificity across connective tissue diseases, including SS. However, higher titers of anti‐Ro52 are positively associated with more aggressive disease, as indicated by the main clinical, immunological, and histopathological features of SS [36]. Anti‐Ro52 antibodies have been demonstrated to be associated with congenital heart block [37] and other autoimmune diseases [13], especially IIM [12, 38]. Concomitant positivity for anti‐Ro52 and antimelanoma differentiation‐associated protein 5 (anti‐MDA5) is frequently observed in dermatomyositis patients with ILD and has been associated with a more severe and rapidly progressive disease course compared with patients who are positive for anti‐MDA5 alone [14]. In addition, a recent cohort study demonstrated an increased risk of ILD progression and lung transplantation or death among patients with ILD who were positive for positive anti‐Ro52 antibodies during a median follow‐up of 25.6 months [39]. Collectively, these findings suggest that anti‐Ro52 antibodies are clinically relevant for risk stratification and prognosis in various disorders. Although anti‐Ro52 antibodies are frequently found in combination with anti‐Ro60 antibodies, especially in SS, separate detection is advisable due to differences in their clinical significance. Notably, our recent multicenter study performed based on the Chinese Sjögren’s Syndrome Collaborative Research Group showed that anti‐Ro60 and anti‐Ro52 antibodies were being detected and reported separately in all the included centers [28]. Therefore, future studies are warranted to incorporate anti‐Ro52 antibody detection into CLIA platforms.

Beyond analytical performance, practical considerations influence assay selection. CLIA and MBI are fully automated, random‐access platforms with minimal hands‐on time and rapid turnaround, while MBI additionally enables true multiplexing from small sample volumes. In contrast, LIA requires manual processing and subjective interpretation, limiting high‐throughput applicability. Although CLIA and MBI improve efficiency and support standardized workflows, these advantages must be balanced against higher costs. Collectively, assay choice should therefore reflect laboratory volume, resources, and the need for quantitative results versus maximal sensitivity.

There are some limitations in the present study. First, this is a cross‐sectional study, and the dynamic profiles of anti‐Ro and anti‐La in the patients have not been repeated. Second, anti‐Ro60 antibodies and/or anti‐La antibodies are present in ~70% of patients with pSS [40]. In the present study, although the patients diagnosed with pSS were randomly recruited from 21 different hospitals, seronegative patients accounted for 7.1% of the included pSS patients probably due to the selection bias of patients. Nevertheless, this study is to compare the analytical performance of automated quantitative assays to that of LIA. Therefore, it is reasonable to speculate that the conclusions would not be affected by the selection bias of pSS patients included in this study. Third, there were some samples reaching the upper detection limit using CLIA. However, it would not affect the specificity and the sensitivity of CLIA in this study. Notably, previous studies have revealed that the titers of anti‐Ro antibodies were significantly associated with the clinical features of SS [36]. Therefore, the samples reaching the upper detection limit should be diluted further to obtain the exact titers of anti‐Ro and anti‐La antibodies in order to explore the association of antibody titers and disease activity as well as the responsiveness of treatment in patients with SS during follow‐up in the longitudinal studies. In addition, this study has solely included patients with pSS and healthy controls in analyses, without including disease controls as the comparators. It is better to include SS‐like patients and other rheumatic disease controls simultaneously as the comparators to obtain more robust results in this study. A further limitation for quantitative comparison arises from the use of different, nonharmonized units across platforms (OD for LIA, AU/mL for CLIA, and AU for MBI), which hampers direct numerical comparisons of antibody levels and emphasizes the need for standardized calibration strategies. Finally, anti‐Ro52 antibodies were not detected using CLIA, because most CLIA test kits in China are solely used for the detection of anti‐Ro60 antibodies. Therefore, the comparison between CLIA and the other assays in the diagnostic value for anti‐Ro52 should be further performed. Notably, certain studies have suggested that combining two different detection assays may increase diagnostic performance [31]. Further cost–benefit studies are needed to address the issue of higher costs from combined assays.

## 5. Conclusions

This multicenter analytical comparison study revealed that automated quantitative assays, CLIA and MBI, are viable alternatives to LIA in the detection of anti‐Ro and anti‐La antibodies in patients with pSS. While MBI showed the highest accuracy, LIA demonstrated superior sensitivity for anti‐Ro60 and anti‐Ro52 detection. CLIA offered reliable specificity but was limited by its inability to detect anti‐Ro52 in this study. The findings support the analytical feasibility of implementing automated quantitative systems for antibody profiling. Nevertheless, further studies incorporating disease controls are required to establish their full clinical utility, particularly for differential diagnosis.

## Author Contributions

Yu‐Lan Chen and Chao‐Jun Hu collected the samples, performed the detection of antibodies, and drafted the manuscript. Lin‐Yi Peng, Dong Xu, and Wen Zhang acquired and analyzed the data. Yan Zhao and Dong‐Zhou Liu involved in the conception and design the study, interpreted the results, and revised the manuscript.

## Funding

This work was funded by Shenzhen Science and Technology Program (Grant JCYJ20240813104215020), the National Natural Science Foundation of China (Grant 81971464), the National Key Research and Development Program of China (Grant 2019YFC0840603), and the Chinese Academy of Medical Sciences Initiative for Innovative Medicine (Grant 2017‐I2M‐3‐001).

## Disclosure

All authors agree to be accountable for all aspects of the work. All authors gave their consent for publication.

## Conflicts of Interest

The authors declare no conflicts of interest.

## Supporting Information

Additional supporting information can be found online in the Supporting Information section.

## Supporting information


**Supporting Information** Table S1. Clinical characteristics of pSS patients. This file contains the demographic, clinical, and laboratory characteristics of the patients with pSS. Table S2. True and false results of each assay in pSS patients. This file includes the detailed numbers of true‐positive, false‐positive, true‐negative, and false‐negative results for each assay in patients with pSS.

## Data Availability

Data underlying the article are available upon reasonable request to the corresponding author.
