# Supplementary material for: Multicenter Analytical Comparison of Automated Quantitative Assays Versus Line Immunoassay for Anti‐Ro/La Antibody Detection in Primary Sjögren’s Syndrome
Source: J Immunol Res. 2026 Jun 19;2026:9771858. doi: 10.1155/jimr/9771858 (PMC13282459; doi:10.1155/jimr/9771858)
Supplement: Supplementary file 1 — Supporting Information Table S1. Clinical characteristics of pSS patients. This file contains the demographic, clinical, and laboratory characteristics of the patients with pSS. Table S2. True and false results of each assay in pSS patients. This file includes the detailed numbers of true‐positive, false‐positive, true‐negative, and false‐negative results for each assay in patients with pSS. [file JIMR-2026-9771858-s001.docx]

**Supplementary Table 1 Clinical characteristics of pSS patients**

| **Variables^£^** | **pSS patients** |
| --- | --- |
| Female, % | 97.4% |
| Age, median (IQR), years | 49.5 (39.0-57.3) |
| Disease duration, months | 24.0 (8.0-58.5) |
| **Clinical features, %** |  |
| Xerostomia | 56.7% |
| Xerophthalmia | 70.5% |
| Parotid swollen | 16.4% |
| Extra-glandular involvement |  |
| Interstitial lung disease | 9.7% |
| Hematological involvement | 35.8% |
| Neurological involvement | 4.5% |
| Renal involvement | 4.1% |
| **Laboratory examinations** |  |
| Positive ANA, % | 93.8% |
| Positive anti-Ro60 antibody, % | 93.6% |
| Positive anti-Ro52 antibody, % | 71.6% |
| Positive anti-La antibody, % | 52.3% |
| Positive RF, % | 38.4% |
| IgG, median (IQR), g/L | 15.9 (13.0-19.9) |
| IgG≥18.0 g/L | 34.7% |
| ESSDAI score | 2.0 (0-7.0) |

Quantitative data are expressed as median (IQR). Categorical variables are expressed as percentages.

ANA: antinuclear antibody; ESSDAI: EULAR Sjogren’s Syndrome Disease Activity Index; IgG: immunoglobulin G; IQR: interquartile range; pSS: primary Sjögren’s syndrome; RF: rheumatoid factor.

^£^ Patients with pSS who had available ESSDAI scores (n=268).

**Supplementary Table 2 True and false results of each assay in pSS patients**

| **Parameter** | **Anti-Ro60** | | | **Anti-Ro52** | | **Anti-La** | | |
| --- | --- | --- | --- | --- | --- | --- | --- | --- |
|  | **LIA** | **CLIA** | **MBI** | **LIA** | **MBI** | **LIA** | **CLIA** | **MBI** |
| **TP** | 402 | 382 | 369 | 387 | 368 | 209 | 221 | 218 |
| **FP** | 0 | 0 | 0 | 5 | 1 | 0 | 0 | 1 |
| **TN** | 100 | 100 | 100 | 95 | 99 | 100 | 100 | 99 |
| **FN** | 32 | 52 | 65 | 47 | 66 | 225 | 213 | 216 |

LIA: line immunoassay; CLIA: chemiluminescence assay; MBI: multiplexed bead immunoassay; TP: true positive; FP: false positive; TN: true negative; FN: false negative.
